# Mindfulness-Based Stress Reduction for Residents: A Randomized Controlled Trial

**DOI:** 10.1007/s11606-017-4249-x

**Published:** 2017-12-18

**Authors:** Hanne Verweij, Hiske van Ravesteijn, Madelon L. M. van Hooff, Antoine L. M. Lagro-Janssen, Anne E. M. Speckens

**Affiliations:** 1 Department of Psychiatry, Radboud University Medical Center, Nijmegen, The Netherlands; 2 Behavioural Science Institute, Radboud University, Nijmegen, The Netherlands; 3 Department of Primary and Community Care, Unit Gender and Womens’ Health, Radboud University Medical Center, Nijmegen, The Netherlands

**Keywords:** emotional exhaustion, burnout, residents, mindfulness, randomized controlled trial

## Abstract

**Background:**

Burnout is highly prevalent in residents. No randomized controlled trials have been conducted measuring the effects of Mindfulness-Based Stress Reduction (MBSR) on burnout in residents.

**Objective:**

To determine the effectiveness of MBSR in reducing burnout in residents.

**Design:**

A randomized controlled trial comparing MBSR with a waitlist control group.

**Participants:**

Residents from all medical, surgical and primary care disciplines were eligible to participate. Participants were self-referred.

**Intervention:**

The MBSR consisted of eight weekly 2.5-h sessions and one 6-h silent day.

**Main Measures:**

The primary outcome was the emotional exhaustion subscale of the Dutch version of the Maslach Burnout Inventory–Human Service Survey. Secondary outcomes included the depersonalization and reduced personal accomplishment subscales of burnout, worry, work–home interference, mindfulness skills, self-compassion, positive mental health, empathy and medical errors. Assessment took place at baseline and post-intervention approximately 3 months later.

**Key Results:**

Of the 148 residents participating, 138 (93%) completed the post-intervention assessment. No significant difference in emotional exhaustion was found between the two groups. However, the MBSR group reported significantly greater improvements than the control group in personal accomplishment (*p* = 0.028, *d* = 0.24), worry (*p* = 0.036, *d* = 0.23), mindfulness skills (*p* = 0.010, *d* = 0.33), self-compassion (*p* = 0.010, *d* = 0.35) and perspective-taking (empathy) (*p* = 0.025, *d* = 0.33). No effects were found for the other measures. Exploratory moderation analysis showed that the intervention outcome was moderated by baseline severity of emotional exhaustion; those with greater emotional exhaustion did seem to benefit.

**Conclusions:**

The results of our primary outcome analysis did not support the effectiveness of MBSR for reducing emotional exhaustion in residents. However, residents with high baseline levels of emotional exhaustion did appear to benefit from MBSR. Furthermore, they demonstrated modest improvements in personal accomplishment, worry, mindfulness skills, self-compassion and perspective-taking. More research is needed to confirm these results.

**Electronic supplementary material:**

The online version of this article (10.1007/s11606-017-4249-x) contains supplementary material, which is available to authorized users.

## INTRODUCTION

Residency is a particularly demanding and challenging period, and a peak time for distress.^1^^,^ 2 Residents are confronted with a high workload and stressful situations. There is a great deal of responsibility for patients, but often a lack of control and autonomy.^3^This can lead to burnout, especially when combined with personal characteristics such as perfectionism, self-judgment and poor emotional regulation.^4^ Burnout is defined as a work-related syndrome characterized by three principal components: emotional exhaustion, depersonalization and reduced personal accomplishment.^4^ In the Netherlands, approximately one fifth of residents have moderate to severe burnout symptoms.^5^ In other countries, these numbers are even higher, ranging from 27% to 82%.^1^^,^ 6 ^,^ 7 Burnout can lead to depression, suicidal thoughts, suboptimal patient care and medical errors.^8^^–^ 11 Given its considerable impact on both their own well-being and the quality of patient care, it is worrying that physicians often do not seek professional help for themselves.^12^

Research on interventions to prevent or reduce resident burnout is scarce.^2^ A review and meta-analysis indicated that cognitive, behavioral and mindfulness-based approaches may contribute to lower levels of burnout among physicians.^13^ In fact, mindfulness has been reported to be helpful not only in reducing burnout but also in promoting well-being and quality of patient care among healthcare professionals.^13^^–^ 15

Mindfulness is defined as intentionally paying attention to the present moment in a non-judgmental way.^16^ Its application as an intervention within healthcare emerged in the 1970s with the 8-week group-based Mindfulness-Based Stress Reduction (MBSR) program. MBSR was initially developed for patients with chronic somatic conditions but is currently offered more broadly, including both patients and healthy individuals.^16^ Although mindfulness has been taught for centuries as part of Buddhist tradition, the meditation practices taught in MBSR are psycho-educational and secular.^16^^,^ 17 MBSR has been found to reduce symptoms of depression and anxiety and to improve quality of life in patients with a variety of somatic and psychiatric disorders.^18^^–^ 20 Several reviews indicate that mindfulness may also reduce burnout and increase well-being in healthcare professionals. More high-quality research is needed though, as the included studies often used limited sample sizes and uncontrolled study designs.^14^^,^ 21 ^,^ 22 In medical students, a few randomized controlled trials have been conducted showing improvements in psychological distress, self-compassion, well-being and empathy.^23^^–^ 25 Burnout was often not assessed in these student populations.

The aim of the current study was to examine the effectiveness of MBSR among residents by conducting a powered randomized controlled trial. We hypothesized that MBSR would significantly reduce burnout compared to the control condition. We also hypothesized that MBSR would reduce worry, work–home interference and medical errors, and increase mindfulness skills, self-compassion, positive mental health and empathy.

## METHODS

### Trial Design

We used a randomized controlled design to compare MBSR with a waitlist control group. The waitlist control group continued with their standard residency and received no intervention during the 3-month control period. After the control period, they were given the opportunity to participate in the MBSR training. The trial was registered at Trialregister.nl, no. NTR4180.

### Participants

The study population consisted of residents from all medical, surgical and primary care disciplines of the Radboud University Medical Center Nijmegen, the Netherlands. The total number of residents varies over time, as their training schedule requires them to rotate to other departments and/or hospitals every 6 to 12 months. However, approximately 1200 residents worked in one of the medical, surgical or primary care disciplines. Residents from all stages of residency were eligible for inclusion as long as their term of residency would not have been expired at baseline assessment. We excluded residents who had participated in an MBSR course previously.

### Procedure

We informed residents about this study on the effects of MBSR on burnout, well-being and patient care during the obligatory introduction days at the beginning of their residency and through monthly newsletters on educational training courses for residents. Residents were self-referred, and when they expressed interest, they were informed in more detail about the study, after which consent was obtained. Residents were able to use educational vouchers or educational budget for participating in the MBSR and therefore did not have to pay themselves. Participants received a certificate for participation to be included in their training portfolio. No further incentives (such as money or course credit) were given. Participants completed an online baseline assessment and were randomized to either the MBSR or the control group. Approximately 3 months later, after the last MBSR session or at the end of the control period, participants received the post-intervention questionnaire. Inclusion and baseline assessment lasted from October 2013 to October 2015. The Medical Ethical Committee Arnhem/Nijmegen, the Netherlands, deemed the study proposal exempt from review, as it did not concern medical-scientific research because of the non-medical nature of the intervention in this population and the minimal risk and burden for participants. Participation was completely voluntary. Residents were not required to inform their educators of their participation, although they were of course allowed to.

### Intervention

Our training used the guidelines of the original MBSR program consisting of eight weekly 2.5-h sessions in the evening and a 6-h silent day during the weekend.^26^ Participants practiced formal mindfulness exercises including the body scan, yoga, and sitting and walking meditation. They received psycho-education about stress, and were instructed to practice daily at home for 45 min. Residents learned to focus their attention on the present moment and observe their own thoughts, feelings and behavior in a kind and non-judgmental way, rather than identifying with them (meta-awareness). They were encouraged to become aware of their own automatic behavior patterns and to consider replacing them with more helpful behavior. The Online Appendix provides a detailed description of the intervention.

Residents participated in regular MBSR courses that were offered by the Radboud Centre for Mindfulness about three evenings a week, four times a year. Group size varied from 8 to 16 participants. The MBSR courses were taught by 11 different trainers, all of whom met the requirements of the good-practice guidance for teaching mindfulness-based courses.^27^ In line with previous studies, completers were defined as having attended four or more MBSR sessions.^28^

### Assessments

#### Primary Outcome

Our primary outcome measure was *emotional exhaustion*, as assessed with the validated Dutch version of the Maslach Burnout Inventory–Human Service Survey, designed to measure burnout in professionals in the human services.^29^^,^ 30 The Dutch version was renamed the Utrecht Burnout Scale (UBOS-C), and two items were deleted because they did not fit well in the proposed factor structure.^30^ The questionnaire measures three burnout components: emotional exhaustion (8 items, α = 0.89), depersonalization (5 items, α = 0.69) and reduced personal accomplishment (7 items, α = 0.79). Emotional exhaustion is seen as the core component. Items were scored on a seven-point scale. *Depersonalization* and *reduced personal accomplishment* were secondary outcome measures.

#### Secondary Outcomes

We used the validated Penn State Worry Questionnaire to measure *worry.* 31 This questionnaire consists of 16 items (α = 0.93), which are scored on a five-point scale. We assessed *negative work–home interference* and *negative home–work interference* with the validated Survey Work–home Interaction NijmeGen.^32^ Negative work–home interference (8 items, α = 0.84) and negative home–work interference (4 items α = 0.74) both use a four-point scale. We measured *mindfulness* skills with the validated Five-Facet Mindfulness Questionnaire Short Form, which consists of 24 items (α = 0.87) scored on a five-point scale.^33^ We used the validated Self-Compassion Scale Short Form to measure *self-compassion* (α = 0.88).^34^ This scale consists of six positively and six negatively worded items, which are scored on a five-point scale, with the former representing self-compassion (α = 0.82) and the latter representing self-criticism (α = 0.85).^35^ We assessed *positive mental health* with the validated Mental Health Continuum–Short Form.^36^^,^ 37 The scale consists of 14 items (α = 0.87), which are scored on a six-point scale. We used the validated Jefferson Scale of Physician Empathy to measure *empathy* in the physician-patient relationship.^38^ It uses a seven-point scale and measures three components of empathy: *perspective-taking* (10 items, α = 0.79), *compassionate care* (8 items, α = 0.58) and *standing in the patients’ shoes* (2 items, α = 0.73). We used questions developed by Prins et al.^10^ regarding *medical errors* (6 items, α = 0.69), which are scored on a five-point scale. All questionnaires were selected based on previous studies in similar populations.^5^^,^ 10 ^,^ 14 ^,^ 25 ^,^ 39

### Sample Size

Based on a previous study, we assumed a four-point difference of post-intervention emotional exhaustion between the MBSR and control groups.^39^ Based on an estimated correlation of 0.5 between the baseline and the post-intervention measurement, a power of 80% and a two-sided *t* test with an alpha of 0.05, we would need approximately 81 subjects per arm. Since we planned to incorporate the baseline levels in the analysis [using analysis of covariance (ANCOVA)], we multiplied this number by a design factor of 0.75,^40^ resulting in 60 subjects required per arm, 120 in total. Taking into account a dropout percentage of 25%, we aimed to recruit 160 participants. As the dropout rate was lower than expected, we stopped recruiting at 148.

### Randomization

The coordinating researcher (HV) randomized participants by means of a computer-generated randomization sequence using an independent website specifically designed for the study. The randomization was minimized, taking into account a) the burnout cut-off level for emotional exhaustion (20 or higher), b) gender (male/female) and c) medical specialty group (medical, surgical, supportive, psychiatry or primary care). Minimization is a method that minimizes the imbalance between the groups over a number of prognostic factors. With minimization, the treatment allocated to the next participant enrolled in the trial depends on the characteristics of those participants already enrolled.^41^

### Statistical Methods

Outcome data were analyzed and reported according to the CONSORT guidelines.^42^ We examined baseline differences between the MBSR and control groups and between participants who dropped out and those who remained in the study by means of chi-square and independent samples *t* tests. We performed the analysis of the intervention outcome according to the intention-to-treat (ITT) principle. We conducted secondary per-protocol analysis with participants in the MBSR group who attended four or more sessions. We compared the post-intervention scores between the two groups with ANCOVA analyses, controlling for baseline measurements and minimization criteria. ANCOVA analysis is a common, standard method for RCTs.^43^ For all tests, we used two-sided *p* values with an alpha < 0.05 level of significance. As the analyses of the secondary outcome measures were exploratory, we did not adjust for multiple testing to avoid type II errors.^44^^,^ 45 We calculated Cohen’s *d*-type effect sizes with the adjusted differences between the groups using the pooled standard deviation at baseline. We performed a sensitivity analysis with multiple imputation techniques to estimate missing values.^46^ We conducted exploratory moderation analyses using the following predictors: gender and emotional exhaustion at baseline. Moderation was examined by adding the potential predictor and its interaction with group to the ANCOVA model.

## RESULTS

### Study Population

**Figure 1:**
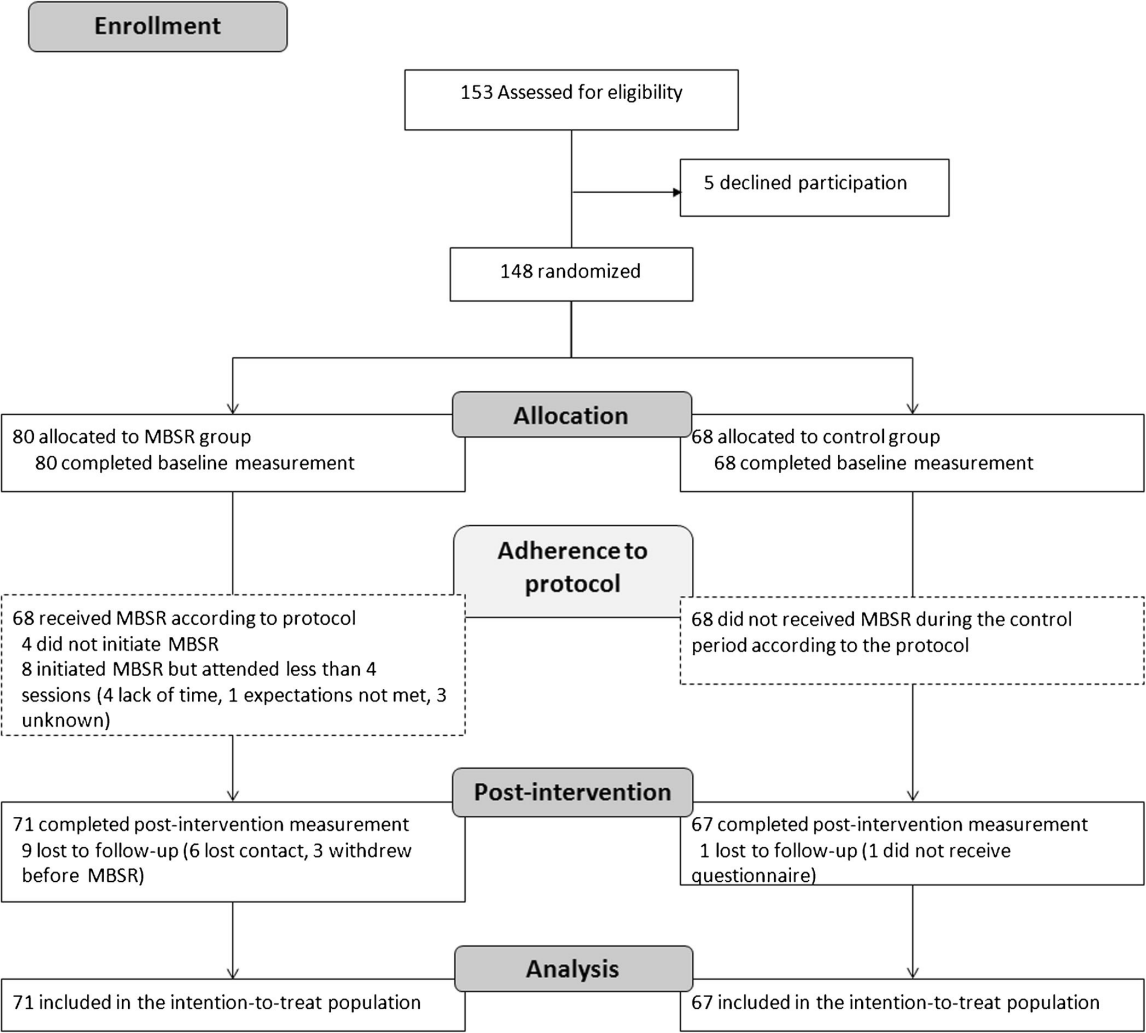
CONSORT flow diagram. Flow of participants through a randomized controlled trial of Mindfulness-Based Stress Reduction, 2013–2016. MBSR = Mindfulness-Based Stress Reduction.

We randomized 148 participants, of whom 138 were ultimately included in the analysis (Fig. 1). There were no baseline differences between the MBSR group and the control group with regard to sociodemographic characteristics (Table 1). However, participants in the MBSR group more often reported work–home and home–work interference than those in the control group (Table 1).

**Table 1:** Baseline Characteristics of Participants in a Randomized Controlled Trial of Mindfulness-Based Stress Reduction, 2013–2016

	Total (*n* = 148)	MBSR (*n* = 80)	Control (*n* = 68)
**Demographic variables, n (%)**			
Female gender	130 (88%)	72 (90%)	58 (85%)
Age, mean (SD)	31.2 (4.6)	31.4 (4.5)	31.0 (4.8)
Marital status			
Married or cohabiting	107 (72%)	54 (68%)	53 (78%)
Partner, not cohabiting	13 (9%)	9 (11%)	4 (6%)
Single	28 (19%)	17 (21%)	11 (16%)
Children			
One or more children	37 (25%)	21 (26%)	16 (23%)
No children	111 (75%)	59 (74%)	52 (77%)
Specialty			
Surgical specialties	17 (11%)	8 (10%)	9 (13%)
Medical specialties	56 (38%)	32 (40%)	24 (35%)
Supportive specialties	18 (12%)	7 (9%)	11 (16%)
Psychiatry	10 (7%)	6 (7%)	4 (6%)
Primary care specialties	47 (32%)	27 (34%)	20 (30%)
Years in training, mean (SD)	2.8 (1.3)	2.8 (1.3)	2.7 (1.4)
Working hours including overtime	46.9 (8.3)	46.7 (8.8)	47.0 (7.7)
**Primary outcome, mean (SD)**			
Burnout – Emotional exhaustion (0–48)	15.6 (7.5)	16.5 (7.8)	14.4 (7.1)
**Secondary outcomes, mean (SD)**			
Burnout – Depersonalization (0–30)	5.2 (3.5)	5.5 (3.9)	4.8 (3.0)
Burnout – Reduced personal accomplishment (0–42)	15.2 (5.0)	15.2 (5.1)	15.1 (5.0)
Worry (16–80)	46.4 (14.2)	48.3 (15.2)	44.0 (12.7)
Work–home interference (1-4)	2.3 (0.4)	2.4 (0.5)	2.2 (0.4) *
Home–work interference (1-4)	1.3 (0.4)	1.4 (0.4)	1.3 (0.3) *
Mindfulness skills (24–120)	74.6 (11.1)	74.5 (11.3)	74.7 (10.9)
Self-compassion (1-7)	3.8 (1.0)	3.9 (1.0)	3.8 (1.1)
*- Positive factor*	*4.1 (1.0)*	*4.1 (1.0)*	*4.1 (1.0)*
*- Negative factor*	*4.4 (1.3)*	*4.4 (1.3)*	*4.5 (1.3)*
Positive mental health (0–5)	3.0 (0.7)	3.0 (0.8)	3.0 (0.7)
Empathy – Perspective-taking (1–7)	5.7 (0.6)	5.7 (0.6)	5.6 (0.7)
Empathy – Compassionate care (1–7)	5.9 (0.7)	5.9 (0.6)	5.9 (0.7)
Empathy – Standing in the patient’s shoes (1–7)	5.8 (0.9)	5.9 (0.9)	5.8 (0.9)
Medical errors (1–5)	2.3 (0.6)	2.4 (0.6)	2.3 (0.5)

MBSR = Mindfulness-Based Stress Reduction

*p < 0.05 for baseline difference between MBSR and control condition (independent samples *t* tests)

Comparing gender of the participants with the total population of residents of the Radboudumc in 2015, the proportion of women among study participants was significantly higher (88% vs. 73%, *p* < 0.001). Participants were also more likely to be trained in one of the medical specialties and less likely from surgical specialties (medical specialties 38% vs. 29%, surgical specialties 11% vs. 17%, supportive specialties 12% vs. 14%, psychiatry 7% vs. 2%, and primary care 32% vs. 37%, *p* < 0.001).

In the MBSR group, 4 (5%) participants did not start MBSR, and 8 (10%) started but did not attend four or more sessions. Non-completers significantly differed from completers in the sense that they more often had children [χ^2^(1) = 7.739, *p* = 0.005], were older [*t*(146) = 2.573, *p* = 0.011], had more negative work–home interference [*t*(146) = 2.291, *p* = 0.023], and scored lower on mindfulness skills [*t*(146) = −2.210, *p* = 0.029], self-compassion [*t*(146) = −2.807, *p* = 0.006] and positive mental health [*t*(146) = −3.210, *p* = 0.002].

### Outcome of the Intervention

Post-intervention emotional exhaustion, our primary outcome measure, did not appear to be lower in the MBSR than in the control group (Table 2). However, regarding the secondary outcome measures, the MBSR group improved in terms of personal accomplishment, worry, mindfulness skills and self-compassion, and on the perspective-taking subscale of the empathy scale compared to the control group. These were small to moderate effects. We found no significant differences in depersonalization, work–home interference, positive mental health, the other components of empathy or medical errors.

**Table 2:** Baseline, Post-Intervention and Group Difference Post-Intervention Scores, from a Randomized Controlled Trial of Mindfulness-Based Stress Reduction, 2013–2016

	Baseline, mean (SD)		Post-intervention, mean (SD)		Post-intervention	*P* value	Cohen’s *d*
	MBSR (*n* = 80)	Control (*n* = 68)	MBSR (*n* = 71)	Control (*n* = 67)	Group difference (95% CI)*		
**Primary outcome**							
Burnout – Emotional exhaustion (0–48)	16.5 (7.8)	14.5 (7.1)	15.0 (5.7)	13.7 (7.8)	0.09 (−1.73 to 1.92)	0.92	0.01
**Secondary outcome**							
Burnout – Depersonalization (0–30)	5.5 (3.9)	4.8 (3.0)	5.1 (3.1)	4.8 (3.1)	0.17 (−0.74 to 1.08)	0.71	0.05
Burnout – Reduced personal accomplishment (0–42)	15.2 (5.1)	15.1 (5.0)	13.9 (4.6)	15.1 (4.5)	−1.19 (−0.13 to −2.25)	0.03	0.24
Worry (16–80)	48.3 (15.2)	44.0 (12.7)	43.1 (14.1)	43.1 (12.9)	−3.21 (−6.20 to −0.21)	0.04	0.23
Work–home interference (1–4)	2.4 (0.5)	2.2 (0.4)	2.2 (0.4)	2.2 (0.4)	−0.08 (−0.19 to 0.03)	0.17	0.18
Home–work interference (1–4)	1.4 (0.4)	1.3 (0.3)	1.3 (0.4)	1.2 (0.3)	0.02 (−0.08 to 0.12)	0.65	0.06
Mindfulness skills (24–120)	74.5 (11.3)	74.7 (10.9)	79.3 (10.3)	76.2 (10.8)	3.61 (0.88 to 6.33)	0.01	0.33
Self-compassion (1–7)	3.9 (1.0)	3.8 (1.1)	4.3 (1.0)	3.9 (1.1)	0.36 (0.09 to 0.63)	0.01	0.35
*- Positive factor*	*4.1 (1.0)*	*4.1 (1.0)*	*4.5 (1.0)*	*4.2 (1.1)*	*0.27 (−0.02 to 0.55)*	*0.07*	*0.26*
*- Negative factor*	*4.4 (1.3)*	*4.5 (1.3)*	*3.9 (1.3)*	*4.4 (1.4)*	*−0.47 (−0.83 to − 0.11)*	*0.01*	*0.36*
Positive mental health (0–5)	3.0 (0.8)	3.0 (0.7)	3.3 (0.6)	3.1 (0.9)	0.08 (−0.12 to 0.28)	0.43	0.10
Empathy – Perspective-taking (1–7)	5.7 (0.6)	5.6 (0.7)	6.0 (0.6)	5.7 (0.6)	0.20 (0.03 to 0.38)	0.03	0.33
Empathy – Compassionate care (1–7)	5.9 (0.6)	5.9 (0.7)	6.0 (0.7)	5.9 (0.8)	0.06 (−0.16 to 0.28)	0.61	0.08
Empathy – Standing in the patient’s shoes (1–7)	5.9 (0.9)	5.8 (0.9)	5.8 (1.0)	5.9 (0.9)	−0.19 (−0.46 to 0.08)	0.16	0.20
Medical errors (1–5)	2.4 (0.6)	2.3 (0.5)	2.3 (0.6)	2.3 (0.6)	−0.05 (−0.20 to 0.10)	0.53	0.09

MBSR = Mindfulness-Based Stress Reduction

*Differences between conditions are adjusted for baseline values

### Per-Protocol Analysis

The per-protocol analysis (*n* = 130) did not reveal large differences compared to the ITT analysis. However, reduced personal accomplishment was not statically significant [group difference = −1.03 (95% CI, 0.09 to −2.14), *p* = 0.072, *d* = 0.20] but statistically significant differences between the MBSR and control group were found in terms of worry [group difference = −3.36 (95% CI, −6.51 to −0.21), *p* = 0.037, *d* = 0.24], mindfulness skills [group difference = 4.22 (95% CI, 1.41 to 7.04), *p* = 0.004, *d* = 0.38], self-compassion [group difference = 0.43 (95% CI, 0.15 to 0.71), *p* = 0.003, *d* = 0.41] and perspective-taking [group difference = 0.20 (95% CI, 0.02 to 0.38), *p* = 0.028, *d* = 0.32].

### Sensitivity Analysis

We conducted a sensitivity analysis using the multiple imputation technique to assess whether missing data affected the outcomes. We found similar improvements of reduced personal accomplishment (pooled difference = −1.22 *p* = 0.049, *d* = 0.24), mindfulness skills (pooled difference = 3.47, *p* = 0.046, *d* = 0.31), self-compassion (pooled difference = 0.35, *p* = 0.012, *d* = 0.33), and perspective-taking (pooled difference = 0.19, *p* = 0.048, *d* = 0.31). Reduction of worry showed a similar effect size but was not significant (pooled difference = −2.97, *p* = 0.083, *d* = 0.21).

### Moderation Analysis

**Figure 2:**
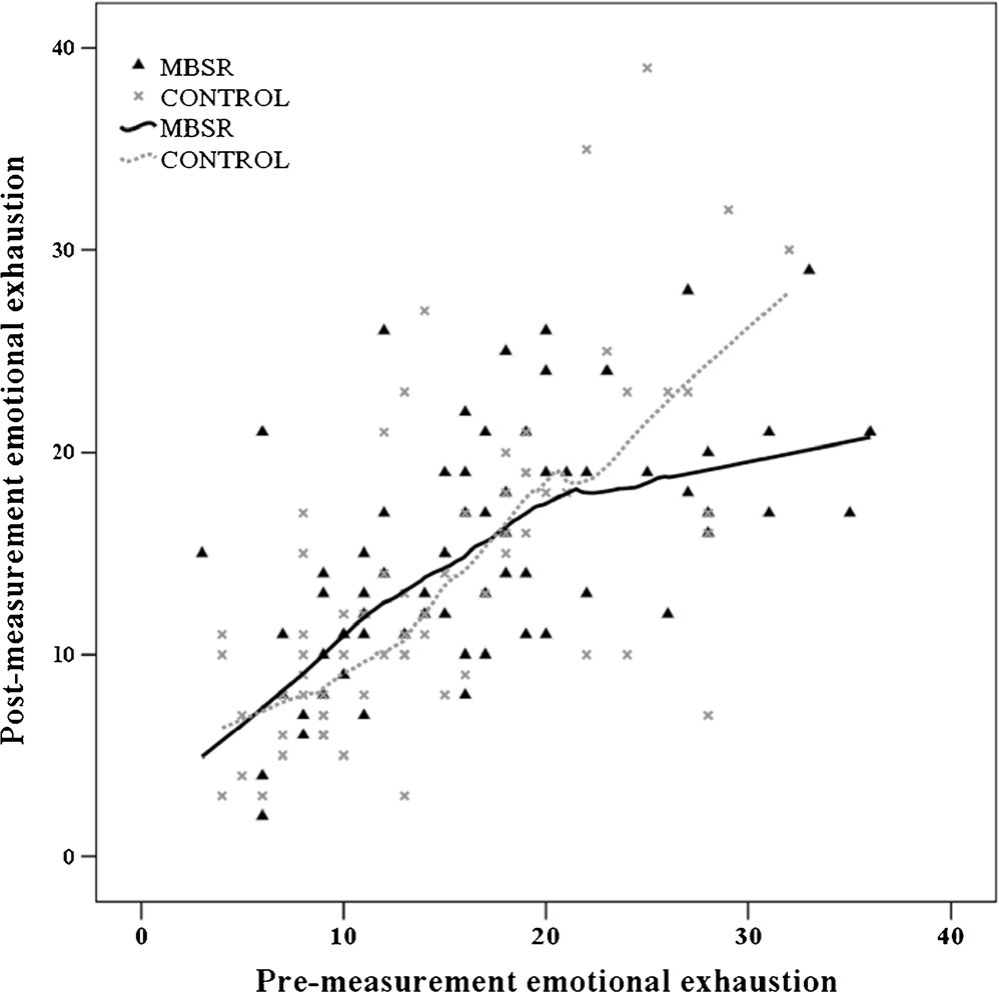
Plot of the for pre- and post-measurement emotional exhaustion scores with Loess curves for both conditions. MBSR = Mindfulness-Based Stress Reduction.

We conducted an exploratory moderation analysis on the primary outcome measure with gender and baseline levels of emotional exhaustion as possible moderators. Gender did not moderate the intervention effect, but baseline levels of emotional exhaustion did [F(1134) = 6.26, *p* = 0.014]. As it appeared that the effect was not completely linear, we fitted a scatterplot with Loess curves to visualize the shape of de moderation effect (Fig. 2).^47^ The reduction of emotional exhaustion as a result of MBSR was dependent on the baseline level of emotional exhaustion: those with higher baseline levels of emotional exhaustion showed greater reductions in emotional exhaustion.

## DISCUSSION

This study is the first randomized controlled trial examining the effectiveness of MBSR in residents. The results of our primary outcome analysis did not support the effectiveness of MBSR in reducing emotional exhaustion among residents. However, baseline levels of emotional exhaustion had a moderating effect on the outcome, indicating that residents with high baseline levels of emotional exhaustion did seem to benefit from the MBSR training. Furthermore, residents participating in the MBSR improved in terms of personal accomplishment, worry, mindfulness skills, self-compassion and perspective-taking (empathy). Therefore, rather than reducing emotional exhaustion, our secondary outcome measures—although preliminary—indicate that MBSR might be beneficial in more general terms of well-being. This is consistent with previous studies on mindfulness in healthy participants.^15^^,^ 24 ^,^ 25 Although the differences between the two groups were modest, they are comparable to those in other studies on MBSR in non-clinical populations.^15^ However, more research is needed to confirm these findings.

In contrast to other studies, however, we did not find an effect on our primary outcome measure.^39^^,^ 48 This might be due to a lower baseline level of emotional exhaustion than expected in our sample, particularly compared with previous studies in U.S. physicians^39^ and Spanish healthcare professionals.^48^ Several meta-analyses on burnout interventions noted that a lack of effect might be explained by a large proportion of participants scoring relatively low on burnout symptoms, leaving limited room for improvement.^49^^,^ 50 Dreison et al.,^49^ for example, found that intervention effects were smaller for samples with lower baseline levels of burnout. Future research may consider examining the effectiveness of MBSR in a population scoring above a particular cut-off for burnout.

It is also possible that reducing burnout in healthcare professionals is particularly difficult. Meta-analyses on burnout interventions in these individuals have found relatively low effect sizes.^49^^,^ 51 However, despite the seemingly modest statistical effect sizes in these studies, a one-point reduction in burnout score can result in clinically meaningful differences.^51^^,^ 52 Unfortunately, the present study was not powered to detect such differences, and this should be further explored in future research.

### Strengths and Limitations

The strengths of our study include its large sample size and real-life setting. The dropout rate of 15% is comparable to that for other MBSR participants and indicates that despite their high workload, for most participating residents the MBSR training was feasible.

There are also some limitations of the study that must be considered. First, the study was performed in a single medical university hospital in the Netherlands, which might limit generalizability. Participants were also self-selected, so the results may have been influenced by selection bias. Men and residents from the surgical specialties were relatively underrepresented. Furthermore, based on our results, we do not know how MBSR compares with alternative active interventions for residents, such as courses in work–life balance or communication skills. The results are also limited to immediate post-intervention outcomes. Further research is needed to investigate the long-term effects of MBSR on residents and its potential for preventing burnout over the long run. Finally, we used participant self-reports to assess changes in empathy and medical errors. Future studies might examine the effect on patient care using patient evaluations or recorded patient visits.

## CONCLUSIONS

Although MBSR did not result in a significant reduction in emotional exhaustion across the group, residents with high baseline levels of emotional exhaustion seemed to benefit from the intervention. This indicates the potential for MBSR as an intervention to address burnout among residents. In addition, MBSR resulted in improvements in personal accomplishment, worry, mindfulness skills, self-compassion and perspective-taking across the study population, suggesting its potential benefit in terms of well-being more generally.

Resident health and well-being have been the focus of increased attention in educational frameworks such as the CanMEDS Physician Competency Framework, the guidelines from the U.S. Accreditation Council for Graduate Medical Education and the UK General Medical Council.^53^^–^ 55 Offering MBSR to residents might be a valuable option to support these efforts. However, our findings are preliminary and should be interpreted with care. More research is needed in order to confirm these findings. In addition, further research could help to determine whether mindfulness should be offered to all or a subset of residents, at what stage of their training and in which format.

## Electronic supplementary material

ESM 1 (DOCX 14 kb)
