# Supplementary material for: Mindfulness-Based Stress Reduction for Residents: A Randomized Controlled Trial
Source: J Gen Intern Med. 2017 Dec 18;33(4):429–36. doi: 10.1007/s11606-017-4249-x (PMC5880763; doi:10.1007/s11606-017-4249-x)
Supplement: Supplementary file 1 — (DOCX 14 kb) [file 11606_2017_4249_MOESM1_ESM.docx]

**Online Appendix**

**Information on Mindfulness-Based Stress Reduction Training (MBSR)**

*Curriculum*

- Developed by Kabat-Zinn^15^ for use in patients with unexplained somatoform disorders
- Eight-week, 2.5-hour training with a fixed program
- Comprehensive folder containing an explanation of the exercises and home practice
- Formal exercises comprise body scan, sitting meditation, and mindful movement
- Informal exercises include awareness of breathing, awareness of routine activities such as eating, walking, cycling
- One-day silent retreat during the sixth week of the program
- Daily home practice with help of CDs/audio recordings comprising weekly alternating formal and informal exercises such as the body scan, sitting meditation, and awareness of routine activities
- Group dialogue and inquiry oriented around weekly in-session exercises and at-home assignments

*Summary of weekly theme with core exercise*

| Week 1: Recognizing  Automatic Behavior  (the automatic pilot) | • Raisin practice: using all senses to explore an object  • Bodyscan: practice being aware of different parts of the body  • A routine activity with deliberate awareness (e.g., taking a shower) |
| --- | --- |
| Week 2: Influence of Perception | • Bodyscan: practice being aware of different parts of the body  • Sitting meditation: awareness of breathing  • A routine activity with deliberate awareness (e.g., taking a shower) |
| Week 3: Recognizing Boundaries | • Yoga: awareness of movement, respecting physical boundaries  • Pleasant events log: inquiry thoughts, feelings, bodily sensations  • Three-minute breathing space, a short pause during the day |
| Week 4: Awareness of Stress | • Sitting meditation: awareness of breathing, body, sounds, thought & emotions  • Unpleasant events log: inquiry thoughts, feelings, bodily sensations  • Yoga: awareness of movement, respecting physical boundaries |
| Week 5: Mindful Response to Stress | • Sitting meditation: awareness of breathing, body, sounds, thoughts & emotions  • Stressful events log: automatic reaction versus mindful response |
| Week 6: Communication | • Yoga; awareness of movement, respecting physical boundaries  • Walking meditation  • Practice mindful communication + group inquiry |
| Silent day | • Varying meditation exercises  • Silent lunch |
| Week 7: Work–Life Balance | • Sitting meditation: choiceless awareness  • Group dialogue: activities demanding energy and giving energy |
| Week 8: Week 8 Lasts the Rest of Your Life | • Bodyscan: practice being aware of different parts of the body  • Make a plan of action for keeping up your skills |
